# Celastrol-Loaded Liposomal Hydrogel Microneedles for Safe and Effective Treatment of Psoriasis

**DOI:** 10.3390/biomedicines14071488

**Published:** 2026-06-30

**Authors:** Jiayi Li, Xiaoyao Fu, Zhonghuan Qu, Yanjun Yang

**Affiliations:** 1 UCL School of Pharmacy, Faculty of Life Sciences, University College London, London WC1E 6BT, UK; lijiayi040627@sina.com; 2 School of Traditional Chinese Pharmacy, China Pharmaceutical University, Nanjing 211198, China; 3225020765@stu.cpu.edu.cn (X.F.); zhq19980221@163.com (Z.Q.)

**Keywords:** celastrol, liposomes, hydrogel microneedles, psoriasis, IL-23/IL-17 axis

## Abstract

**Background**: Psoriasis is a chronic inflammatory skin disease characterized by abnormal epidermal hyperplasia and immune-inflammatory imbalance. Although celastrol (Cel) exhibits potent anti-inflammatory activity, its strong hydrophobicity and low local delivery efficiency limit its therapeutic application. **Methods**: To enhance its transdermal delivery and topical therapeutic efficacy, a Cel-loaded liposomal hydrogel microneedle system (Cel-lipo-MNs) was developed in this study. Cel-loaded liposomes were first prepared by the thin-film dispersion method, and Cel-lipo-MNs were subsequently fabricated using a multistep vacuum micromolding process combined with UV-induced photocrosslinking. **Results**: In vivo studies demonstrated that, in an imiquimod-induced psoriasis-like mouse model, Cel-lipo-MNs markedly alleviated erythema, scaling, and skin thickening, reduced PASI-like scores. Further investigation revealed that Cel-lipo-MNs significantly downregulated the serum levels of IL-17, IL-23, and IFN-γ, and exhibited superior therapeutic efficacy compared with free celastrol, conventional liposomes, and blank microneedles. **Conclusions**: These findings indicate that Cel-lipo-MNs can substantially enhance the therapeutic effect of celastrol against psoriasis-like skin lesions, possibly through suppression of the IL-23/IL-17 inflammatory axis and related immune-inflammatory responses, and provide a promising transdermal delivery strategy for topical psoriasis treatment.

## 1. Introduction

Psoriasis is a chronic, relapsing autoimmune skin disease pathologically characterized by abnormal proliferation and differentiation of keratinocytes, as well as dysregulation of innate and adaptive immunity [1]. More than 60 million people are affected worldwide [2], and the prevalence of adult psoriasis ranges from 0.51% to 11.43% across more than 20 countries, including the United States, Germany, and South Korea [3]. Psoriasis imposes long-term physical suffering, psychological burden, and substantial socioeconomic pressure on patients [4,5,6]. Its pathogenesis is highly complex, among which overactivation of the IL-17/IL-23 inflammatory axis is recognized as a key driver of lesion initiation and progression [7,8]. At present, psoriasis remains incurable, and patients often require long-term maintenance therapy. Thus, the selection of clinical treatment strategies continues to be constrained by the need to balance efficacy and safety [9,10].

Current clinical treatments for psoriasis mainly include topical therapy and systemic therapy [11]. Topical corticosteroids [12] and vitamin D_3_ derivatives [13] are first-line options for mild to moderate psoriasis [14], but the natural barrier of the stratum corneum severely restricts transdermal drug penetration, making it difficult to achieve effective therapeutic concentrations at dermal lesion sites. Long-term use may also induce local adverse effects such as skin atrophy, telangiectasia, and pigmentary abnormalities [15]. Although systemic agents such as methotrexate and cyclosporine can control moderate to severe disease [16], their clinical use is limited by significant toxicities, including gastrointestinal intolerance, hepatorenal injury, and bone marrow suppression [17,18,19]. Therefore, there is an urgent need to develop a novel topical delivery system capable of efficiently overcoming the skin barrier, precisely targeting lesion sites, achieving sustained drug release, reducing systemic adverse effects, and improving patient compliance.

Celastrol (Cel) is a pentacyclic triterpenoid active compound isolated from the traditional Chinese medicinal herb Tripterygium wilfordii Hook. f. [20]. It possesses potent anti-inflammatory [21], immunosuppressive [22], and antiproliferative activities [23], making it a highly promising natural bioactive molecule for psoriasis therapy [24]. Previous studies have demonstrated that Cel can directly bind to IL-17A and block its downstream NF-κB and MAPK inflammatory signaling pathways, thereby markedly alleviating psoriatic lesions in mouse models [25]. However, its extremely poor water solubility [26], low bioavailability [27], and multi-organ toxicity [28] have become major barriers to clinical translation. Therefore, it is necessary to optimize its formulation in order to fully exploit its therapeutic potential.

Advances in nanocarriers and microneedle-based delivery technologies provide new opportunities to address these challenges. Unlike polymeric nanoparticles or solid lipid nanoparticles, the phospholipid bilayer structure of liposomes closely mimics the architecture of biological membranes, thereby facilitating intimate contact with the stratum corneum and promoting lipid exchange and fluidization at the skin barrier interface [29]. Furthermore, the compositional versatility of liposomes permits the incorporation of functional excipients—such as cholesterol for membrane stabilization [30] and PEGylated lipids for steric stabilization [31,32]. In addition, PEGylation also contributes to the biocompatibility of nanocarrier systems [33]. demonstrated that PEG modification significantly enhanced the dispersibility of PDA nanoparticles while reducing their cytotoxicity toward HaCaT cells. These findings suggest that PEGylation not only stabilizes nanosystems but also improves their biosafety.

Hydrogel microneedles, as a minimally invasive transdermal delivery platform, can mechanically penetrate the stratum corneum with micron-scale needle arrays [34], directly bypass the skin barrier, and precisely deliver drugs into dermal lesion sites, thus overcoming the poor transdermal efficiency of conventional topical formulations [35]. Microneedles and liposomes have a potential synergistic effect in enhancing the penetration of transdermal drug delivery systems [36].

Based on these considerations, the present study aimed to construct a celastrol-loaded liposomal hydrogel microneedle delivery system. Through optimization of formulation composition and preparation process, systematic pharmaceutical characterization was performed, including particle size, encapsulation efficiency, mechanical performance, and transdermal properties. Furthermore, using an imiquimod-induced psoriasis mouse model, the therapeutic efficacy of this delivery system against psoriasis-like lesions was evaluated. This study is expected to provide an efficient, safe, and patient-compliant strategy for the topical treatment of psoriasis, as well as new experimental evidence and technical support for the clinical translation of Cel in chronic inflammatory skin diseases.

## 2. Materials and Methods

### 2.1. Materials

Celastrol (Cel, CAS: 34157-83-0, purity ≥ 98%) was purchased from Nanjing Jingzhu Biotechnology Co., Ltd. (Nanjing, China). Distearoyl phosphatidylethanolamine-polyethylene glycol 2000 (DSPE-PEG2000, CAS: 147867-65-0) and 1,2-dioleoyl-sn-glycero-3- phosphocholine (DOPC, CAS: 4235-95-4) were obtained from Shanghai Yuanye Biotechnology Co., Ltd. (Shanghai, China). Cholesterol (purity > 95%), polyethylene glycol diacrylate (PEGDA, average molecular weight 1000, CAS: 26570-48-9), acrylamide (AM, CAS: 79-06-1), and 2-hydroxy-4’-(2-hydroxyethoxy)-2-methylpropiophenone (I2959, CAS: 106797-53-9) were supplied by Shanghai Aladdin Biochemical Technology Co., Ltd. (Shanghai, China). PDMS microneedle molds (height: 600 µm, base diameter: 300 µm, array: 20 × 20) were purchased from Anhui Zhongding Yuxuan New Material Technology Co., Ltd. (Hefei, China). 5% imiquimod cream was obtained from Hubei Keyi Pharmaceutical Co., Ltd. (Wuhan, China), and compound dexamethasone acetate cream was purchased from China Resources Sanjiu Medical & Pharmaceutical Co., Ltd. (Shenzhen, China). ELISA kits for IL-17, IL-23, and IFN-γ were purchased from Jiangsu Enzyme Immuno Industrial Co., Ltd. (Yancheng, China). All other reagents used were of analytical grade.

### 2.2. Animals

A total of 42 healthy SPF-grade male BALB/c mice (6 weeks old, weighing 18–22 g) were purchased from Zhejiang Vital River Laboratory Animal Technology Co., Ltd. (license No. SYXK (Su) 2023-0019, Pinghu, China). The animals were housed under barrier conditions at a room temperature of 25 °C with a 12 h light/dark cycle and were allowed free access to food and water. All animals were maintained in accordance with relevant laws and regulations, and humane care was provided in compliance with the principles of the 3Rs.

### 2.3. Preparation and Characterization of Cel-Lipo

Celastrol-loaded liposomes (Cel-lipo) were prepared using the thin-film dispersion method. Briefly, Cel, DOPC, cholesterol, and DSPE-PEG2000 were completely dissolved in a certain volume of chloroform at a specific mass ratio, transferred into a round-bottom flask, and then subjected to rotary evaporation under reduced pressure to slowly remove the organic solvent, resulting in the formation of a uniform and transparent lipid film on the inner wall of the flask. Thereafter, PBS buffer with a pH of 7.4 ± 0.2 was added, and the film was hydrated at 60 °C for 30 min, followed by sonication for 10 min (15 s on, 15 s off, ultrasonic power was set to 60%, instrument was purchased from Shanghai Bilang Instrument Manufacturing Company (Shanghai, China)). Finally, the resulting suspension was filtered through a 0.22 μm microporous membrane and adjusted to 5 mL with PBS buffer to obtain Cel-lipo, which was stored at 4 °C.

**Figure A1:**
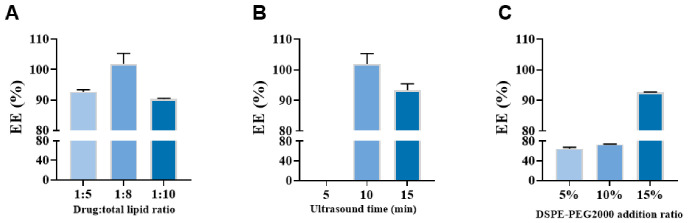
Optimization of the Cel-Lipo formulation. (**A**). Effect of drug-to-total lipid mass ratio on the EE% of liposomes. (**B**). Effect of ultrasonication time on the EE% of liposomes. (**C**). Effect of DSPE-PEG2000 content on the EE% of liposomes.

Further optimization of the Cel-lipo formulation was carried out using encapsulation efficiency (EE%) as the response value. The amount of Cel encapsulated in the Cel-lipo formulation was determined by ultrafiltration. Briefly, 0.5 mL of the liposomal suspension was subjected to ultrafiltration, and the filtrate containing the free drug was collected for quantitative analysis. In parallel, another 0.5 mL aliquot of the liposomal suspension was disrupted by sonication to determine the total Cel content, from which the EE% was calculated. Single-factor experiments were performed with the drug-to-total-lipid mass ratio (1:5, 1:8, and 1:10), DSPE-PEG2000 content (5%, 10%, and 15%), and sonication time (5, 10, and 15 min) as independent variables, by which the optimized formulation of Cel-lipo was obtained. The specific optimization results can be found in Appendix A (Figure A1). Based on the formulation optimization experiments, the optimal mass ratio of DOPC:cholesterol:Cel:DSPE-PEG2000 was determined to be 6:1:0.875:1.05. The total lipid concentration of the liposomal formulation was 9.68 mg/mL. The drug loading capacity (LC%) represents the percentage of drug mass relative to the total mass of the liposomal formulation. Briefly, the amount of Cel encapsulated in the Cel-lipo formulation was determined according to the method used for EE% determination described above, while the total mass of the liposomal formulation was obtained after freeze-drying.

The particle size, polydispersity index (PDI), and zeta potential of Cel-lipo were measured using a Zetasizer Nano ZS90 (Malvern Instruments, Malvern, UK, Scattering angle: 90°). In addition, the stability of Cel-lipo stored at 4 °C for 0, 7, 14, 21, and 28 days was evaluated in terms of appearance and particle size and PDI.

### 2.4. Preparation and Characterization of MNs

Photocrosslinked hydrogel microneedles loaded with Cel-lipo (Cel-lipo-MNs) were prepared using a multistep vacuum micromolding method. The precursor hydrogel matrix was obtained by mixing 0.5 mL of Cel-lipo solution with 0.5 mL of PEGDA solution containing 100 mg of AM and 1% photoinitiator I2959 [37]. A certain volume of the resulting mixture was carefully added into the mold and placed in a vacuum drying oven. The mold was evacuated to −0.08 MPa and maintained under vacuum for 15 min. Afterward, the excess matrix solution on the mold backing was removed, fresh matrix solution was added again, and vacuum was maintained for another 15 min. The filled mold was then exposed to UV light (365 nm, 5 W) for crosslinking to form microneedles. Finally, the microneedle array was carefully peeled off from the reverse mold, placed in a desiccator, and dried overnight until completely dried. Blank microneedles without Cel-lipo (Blank-MNs) were prepared using the same procedure.

Morphological characterization: The morphology of Cel-lipo-MNs was observed using a Canon PowerShot SX130 IS camera and a scanning electron microscope (ZEISS Sigma 360, ZEISS, Oberkochen, Germany). The microneedle samples were fixed onto the specimen stage with conductive adhesive and then sputter-coated with gold. The coated samples were placed in the SEM chamber, and after vacuuming, they were observed in secondary electron mode.

Mechanical strength: The mechanical strength of Cel-lipo-MNs was analyzed using a TMS-Pro texture analyzer [38] (Food Technology Corporation, Sterling, VA, USA). A PA2S probe with a diameter of 2 mm was moved vertically toward the microneedles at a speed of 0.02 mm/s. When the sensor came into contact with the microneedle tips (trigger force reached 0.02 N), displacement and force measurement was initiated, and the speed was adjusted to 0.6 mm/min. The measurement was continued until the probe had moved 500 μm.

Biocompatibility: A hemolysis assay was performed using red blood cells collected from the orbital venous plexus of mice [39]. The blood samples were washed three times with normal saline by centrifugation and then diluted to obtain a 4% red blood cell suspension for testing. Different concentrations of the microneedle matrix were added to the red blood cell suspension. PBS and ultrapure water of equal volume were used as the negative and positive controls, respectively. After incubation at 37 °C for 1 h, the samples were centrifuged at 3000 rpm, and the supernatants were collected. The absorbance was measured at 540 nm using a microplate reader. The hemolysis rate was calculated according to Equation (1).

Fourier transform infrared (FTIR) spectroscopy: The chemical compositions of the prepared Blank-MNs (PEGDA-AM-MNs), as well as PEGDA, and AM, were characterized using an FTIR spectrometer (Bruker TENSOR27, Bruker Corporation, Bremen, Germany). The dried microneedle samples were ground into powder, mixed with dried potassium bromide, and compressed into pellets. The spectra were recorded over a wavenumber range of 4000–500 cm^−1^.

Drug loading: To evaluate the drug loading of Cel in Cel-lipo-MNs, the microneedle array was immersed in 4 mL of water. The solution was stirred overnight at 200 r/min, followed by sonication to ensure complete drug release. Then, 1 mL of the above solution was mixed with 1 mL of methanol and sonicated for 30 min, followed by centrifugation at 12,000 r/min for 10 min. The supernatant was collected, filtered through a 0.22 μm membrane, and analyzed by HPLC to determine the Cel content, from which the drug loading in the microneedles was calculated [40].

### 2.5. Insertion Experiment and Release Behavior of MNs

An ex vivo mouse skin model was used to evaluate the insertion performance of the microneedles. The Cel-lipo-MNs array was pressed into the mouse skin for 2 min. After removal of the microneedle array, the recovery of the skin puncture sites was monitored within 30 min and photographed using a Canon PowerShot SX130 IS camera. For histological analysis, the skin tissue at the microneedle insertion site was excised with a scalpel. The skin samples were then fixed in 4% paraformaldehyde for 24 h, embedded in paraffin, sectioned using a cryomicrotome, and stained with hematoxylin and eosin (H&E). Finally, the sections were observed using an S60 whole-slide scanner (NanoZoomer S60 C13210-01, Hamamatsu Photonics K.K, Shizuoka, Japan). Agarose gel blocks were used to simulate skin tissue in order to evaluate the drug release behavior of PEGDA-AM-based hydrogel microneedles after insertion into the skin [41]. Furthermore, in accordance with the preparation procedure of Cel-lipo-MNs, the Cel-lipo solution was replaced with a 5% phenol red solution to fabricate phenol red-loaded hydrogel microneedles. These microneedles were then inserted into solid agarose, and the release behavior of phenol red was observed.

A transdermal release study was conducted using a Franz diffusion cell system. Phosphate-buffered saline (PBS, pH 7.4) was used as the release medium to evaluate the transdermal release profiles of free Cel, Cel-lipo, and Cel-lipo-MNs. Excised mouse skin was mounted between the donor and receptor compartments of the Franz diffusion cell, with the stratum corneum facing the donor compartment. Cel solution, Cel-lipo suspension, and Cel-lipo-MNs containing approximately 70 μg of celastrol were separately placed in the donor compartment, while the receptor compartment was filled with PBS (pH 7.4). The Franz diffusion cells were maintained at 37 °C with continuous magnetic stirring at 300 rpm. At predetermined time points (0, 2, 4, 6, 8, 12, and 24 h), 0.5 mL of the receptor medium was withdrawn and immediately replaced with an equal volume of fresh PBS. The collected samples, after ultrasonic treatment, were determined by HPLC using an Agilent TC-C18 column (4.6 mm × 250 mm, 5 μm). The mobile phase consisted of 0.1% phosphoric acid aqueous solution and methanol (22:78, *v*/*v*), and isocratic elution was performed for 60 min. The flow rate was set at 1.0 mL/min, the column temperature was maintained at 30 °C, and the injection volume was 10 μL. Cel was detected at a wavelength of 425 nm.

### 2.6. Evaluation of the Therapeutic Efficacy of Cel-Lipo-MNs in an IMQ-Induced Psoriasis Model

The efficacy of the Cel-lipo-MNs delivery system was evaluated in an imiquimod (IMQ)-induced psoriasis-like mouse model [42]. After 5 days of acclimatization, the dorsal hair of the 42 healthy BALB/c mice was shaved and further removed with Veet depilatory cream to obtain an area of approximately 2 cm × 2 cm. The mice were then allowed free access to food for 24 h to allow recovery of the stratum corneum on the dorsal skin. Subsequently, the mice were randomly divided into seven groups (*n* = 6): Control group (0.1 mL saline), Model group (0.1 mL saline), Positive control group (0.1 mg dexamethasone cream/10 g body weight), Cel group (0.1 mL, Cel concentration 0.1 mg/mL), Cel-lipo group (0.1 mL, Cel concentration 0.1 mg/mL), Blank-MNs group, and Cel-lipo-MNs group. A psoriasis-like model was induced by daily topical application of 62.5 mg of 5% IMQ cream for 7 consecutive days, followed by drug administration 6 h later. In addition, body weight was recorded daily, and erythema, scaling, and skin thickness were scored according to the Psoriasis Area and Severity Index (PASI). On day 8, the mice were sacrificed, and blood, skin, and spleen samples were collected for subsequent analysis.

### 2.7. Histological Analysis and Epidermal Thickness

On day 8, skin tissues were collected and fixed in 4% paraformaldehyde. After 24 h, the samples were subjected to H&E staining and histologically examined using an S60 whole-slide scanner. Histopathological features, including capillary proliferation, inflammatory cell infiltration, hyperkeratosis, and acanthosis, were analyzed. In addition, three random fields were selected, and epidermal thickness was measured using ImageJ software (ImageJ 1.54p).

### 2.8. Detection of Spleen Index

Before sacrifice under isoflurane anesthesia, the body weight of each mouse was recorded. After euthanasia, the spleen was carefully excised, blotted to remove residual blood, and accurately weighed. The spleen index was then calculated as the ratio of spleen weight (mg) to body weight (g).

### 2.9. Detection of Serum Cytokine Levels

On day 8, blood samples were collected from the mice and allowed to stand at room temperature for 2–3 h, followed by centrifugation at 3000 r/min for 20 min to obtain the serum from the supernatant. The serum levels of IL-17, IL-23, and IFN-γ were then determined according to the instructions of the enzyme-linked immunosorbent assay (ELISA) kits.

### 2.10. Data Analysis

Statistical analysis was performed using GraphPad Prism version 10.4.1, and the data are presented as mean ± standard deviation (SD). Comparisons between two groups were conducted using the *t*-test, while comparisons among multiple groups were performed using one-way analysis of variance (one-way ANOVA). A value of *p* < 0.05 was considered statistically significant. Abstract image drawn using BioGDP material [43].

## 3. Results

### 3.1. Subsection

#### 3.1.1. Formulation and Characterization of Cel-Lipo

**Figure 1:**
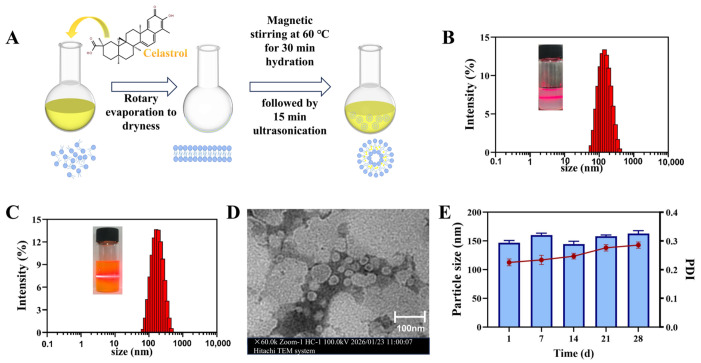
Preparation and characterization of Cel-lipo. (**A**). Schematic diagram of the preparation process of Cel-lipo; (**B**). Particle size distribution of Blank-lipo; (**C**). Particle size distribution of Cel-lipo; (**D**). Particle size of the Cel-lipo when stored in PBS over a period of 28 d; (**E**). PDI stability of the Cel-lipo when stored in PBS over a period of 28 d.

DOPC is the principal film-forming phospholipid in the liposomal system and constitutes the main structural framework of Cel-lipo. Cholesterol serves as a classical membrane stabilizer and regulator, whereas DSPE-PEG2000, as a functional modifying lipid, can form a hydrophilic hydration layer on the liposomal surface, thereby improving the physical and chemical stability of the nanocarrier under physiological conditions [44]. In this study, Cel-lipo was prepared by the thin-film dispersion method (Figure 1A) [45], and its formulation composition was further optimized through single-factor experiments. The optimal mass ratio of DOPC:cholesterol:Cel: DSPE-PEG2000 was determined to be 6:1:0.875:1.05. Under these conditions, the particle size of the prepared Cel-lipo was 146.93 ± 3.85 nm (Figure 1C), which was markedly larger than that of the blank liposomes (126.53 ± 0.49 nm, Figure 1B), suggesting that Cel was successfully encapsulated within the liposomes. The zeta potential of Cel-lipo was relatively low, at −1.83 ± 0.92 mV. The EE% of the Cel was 93.90%, the LC% of the liposomes was 5.166%. Cel is practically insoluble in water, with a reported aqueous solubility of 13.25 ± 0.83 μg/mL at 37 °C [46]. After encapsulation into our liposomes, the Cel concentration achieved in the liposomal dispersion reached 0.470 mg/mL, corresponding to an approximately 35-fold increase in apparent aqueous solubility compared with free Cel.

To further verify the stability of Cel-lipo, its storage stability at 4 °C for one month was investigated. No flocculent precipitate was observed, and the particle size remained around 150 nm (Figure 1D). The PDI on day 0 was 0.2253, and it remained below 0.3 during the monitoring period. (Figure 1E), indicating that the optimized Cel-lipo exhibited good stability. Overall, the Cel-lipo obtained in this study showed a uniform particle size distribution and favorable stability.

#### 3.1.2. Formulation and Characterization of Cel-Lipo-MNs

**Figure 2:**
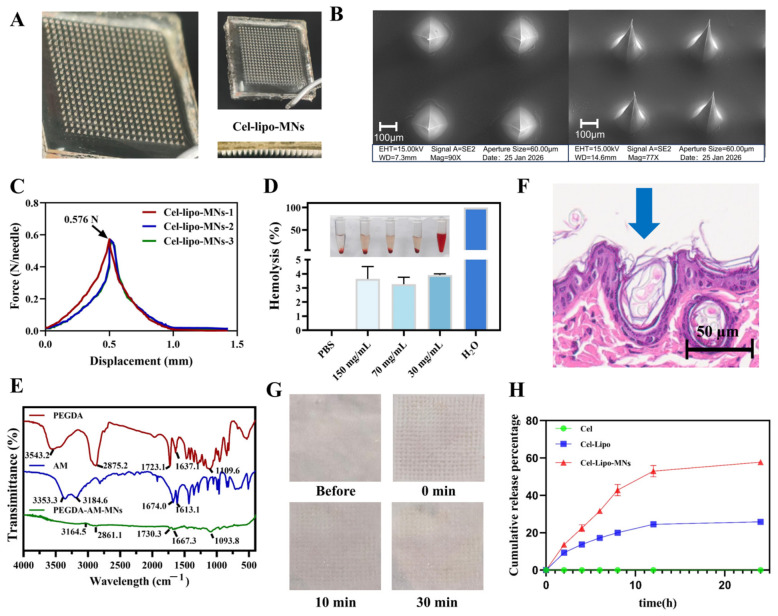
Preparation and characterization of Cel-lipo-MNs. (**A**). Digital camera images of Cel-lipo-MNs; (**B**). SEM top and side view of Cel-lipo-MNs; (**C**). Mechanical strength of Cel-lipo-MNs; (**D**). Hemolysis rates of Cel-lipo-MNs at different concentrations; inset shows images of the hemolysis test; (**E**). Infrared spectra of PEGDA, AM, and PEGDA-AM-MNs; (**F**). H&E staining of mouse skin after application of Cel-lipo-MNs; (**G**). Mouse skin recovery status after application of Cel-lipo-MNs; (**H**). Release behavior of Cel-Lipo-MNs.

In this study, I2959 was used as the photoinitiator, while PEGDA (MW = 1000) and AM, which possess photocrosslinkable capability and good biocompatibility, were selected as the matrix materials for hydrogel microneedles. The microneedles were fabricated by a vacuum micromolding-assisted photopolymerization method, and Cel-lipo was incorporated into the microneedle matrix. Camera and scanning electron microscopy showed that the prepared Cel-lipo-MNs exhibited an intact structure, consisting of a 20 × 20 array of conical needle tips with a tip-to-tip spacing of 600 μm, a height of 600 μm, and a base diameter of 300 μm. The needle tips were sharp and uniformly arranged, with no obvious bending or collapse (Figure 2A,B).

Mechanical strength testing is an important characterization method to evaluate whether microneedles can effectively penetrate the skin barrier and avoid bending or fracture during application. The force-displacement curve showed that the Cel-lipo-loaded AM-PEGDA microneedles could withstand a maximum force of 0.576 N/needle (Figure 2C), which was markedly higher than the minimum mechanical threshold required for skin penetration (0.058 N/needle) [47], indicating that Cel-lipo-MNs possessed good mechanical strength and sufficient skin insertion capability. In addition, the Cel loading in the microneedles was 70.69 ± 2.36 µg.

Microneedles achieve drug delivery by penetrating the stratum corneum. If the materials or leachable components possess potential hemolytic activity, they may come into contact with blood through local microinjury during insertion, thereby posing additional safety risks. Therefore, the hemocompatibility of Cel-lipo-MNs at different concentrations was further evaluated. As shown in the hemolysis assay results (Figure 2D), the suspension in the positive control group (water) appeared distinctly red, and no red blood cell sediment was observed at the bottom of the tube, whereas the suspension in the negative control group (normal saline) was clear and colorless, with obvious red blood cell sediment visible at the bottom. In contrast, the suspensions of all Cel-lipo-MNs groups were colorless or pale in color, and red blood cell sediment was observed at the bottom of each tube, which was generally consistent with the negative control group. Quantitative analysis further showed that the hemolysis rates of all groups were below 5%, indicating that the prepared Cel-lipo-MNs exhibited good hemocompatibility [48].

The photoinitiator I2959 can decompose under 365 nm UV irradiation to generate active free radicals, which attack the acrylate double bonds at the termini of PEGDA and initiate polymerization. As a polymerizable small-molecule monomer, AM can also participate in free radical polymerization, ultimately forming a three-dimensional crosslinked network that converts the fluid precursor solution into hydrogel microneedles with certain mechanical strength. FTIR analysis of the PEGDA-AM-based hydrogel microneedles and their constituent components (Figure 2E) showed that PEGDA exhibited a characteristic ester C=O absorption peak at 1723.1 cm^−1^ and a C=C-related absorption peak of the acrylate group at 1637.1 cm^−1^, indicating the presence of double-bond structures capable of participating in photocrosslinking. AM showed –NH_2_ stretching vibration peaks at 3353.3 and 3184.6 cm^−1^, as well as amide-related characteristic absorption peaks at around 1674.0 and 1613.1 cm^−1^. Compared with PEGDA and AM, PEGDA-AM-MNs displayed shifted characteristic absorption peaks at 3164.5, 1730.3, 1667.3, and 1093.8 cm^−1^, while the characteristic peak associated with the PEGDA double bond was markedly weakened. These results indicate that PEGDA and AM successfully underwent polymerization and crosslinking under photoinitiated conditions, ultimately forming a new PEGDA-AM hydrogel network structure.

#### 3.1.3. Insertion Performance and Release Behavior of Cel-Lipo-MNs

H&E staining results (Figure 2F) showed that Cel-lipo-MNs could create clear microneedle channels in the skin tissue, indicating that they possessed sufficient mechanical strength and favorable skin insertion capability, thereby facilitating the delivery of Cel-lipo to the lesion site and improving their local bioavailability and therapeutic efficacy. In addition, after insertion of Cel-lipo-MNs into ex vivo mouse skin (Figure 2G), a regularly arranged array of micropores was observed on the skin surface, indicating that the microneedles could effectively penetrate the superficial skin layer and form uniform insertion channels. With increasing time, the micropore traces on the skin surface gradually diminished and nearly disappeared within 30 min, suggesting that the skin disruption caused by microneedle insertion was temporary and that the skin exhibited good recovery capability. These results demonstrate that Cel-lipo-MNs can achieve effective transdermal penetration while exerting only a mild impact on the skin barrier, showing favorable minimally invasive and reversible characteristics. In addition, the drug release behavior of the microneedles was evaluated using Franz diffusion cells (Instrument: Shanghai Huanghai Pharmaceutical Inspection Instrument Co., Ltd. (Shanghai, China); Temperature: 37 °C; Rotation speed: 300 rpm.) mounted with ex vivo mouse skin, which demonstrated that the Cel-lipo-MNs exhibited good release capability within 24 h (57.79 ± 0.968%). (Figure 2H), suggesting that the microneedles could achieve drug delivery through water absorption-induced swelling and diffusion within the hydrogel network.

#### 3.1.4. In Vivo Studies on Anti-Psoriatic Effects of Cel-Lipo-MNs

**Figure 3:**
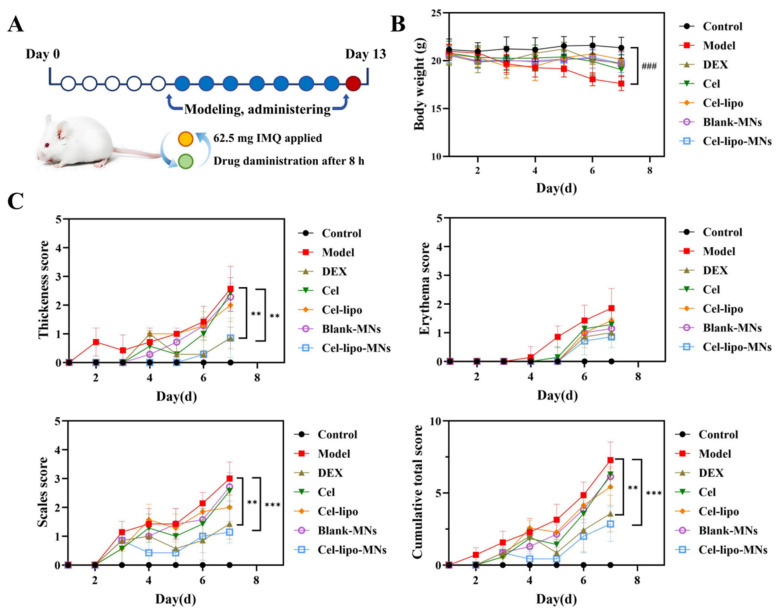
PASI scores and body weight of different groups on psoriasis models. (**A**). Schematic diagram of the establishment and treatment of the IMQ-induced psoriasis model; (**B**). Changes in body weight during treatment; (**C**). PASI scores of psoriatic skin. Data are expressed as mean ± SD (*n* = 6). * or # *p* < 0.05, ** or ## *p* < 0.01, and *** or ### *p* < 0.001. “#” stands for drug group vs. Control group, “*” stands for drug groups vs. Model group.

To evaluate the therapeutic potential of Cel-lipo-MNs against psoriasis, an imiquimod (IMQ)-induced psoriasis-like mouse model and the corresponding treatment regimen were established in this study (Figure 3A). During the modeling and treatment period, the body weight of mice in the model group was significantly reduced compared with that in the control group (*p* < 0.001), whereas no obvious abnormal body weight loss was observed in the other groups (Figure 3B). In addition, obvious lesion progression was observed in the model group from day 3–4 after modeling, as evidenced by continuously increased scores for skin thickening, erythema, and scaling, as well as a marked increase in cumulative PASI-like scores, indicating successful establishment of the model (Figure 3C). Compared with the model group, all treatment groups delayed lesion aggravation to varying extents, among which the Cel-lipo-MNs group showed the most pronounced improvement. This group exhibited lower scores for thickness, erythema, scaling, and cumulative total score than the model group, and overall performed better than the Cel, Cel-lipo, and Blank-MNs groups, showing a therapeutic trend comparable to that of the DEX group. These results indicate that the microneedle-mediated liposomal delivery system can more effectively enhance transdermal drug delivery and improve local therapeutic efficacy, thereby significantly alleviating psoriasis-like lesions and suppressing disease progression.

**Figure 4:**
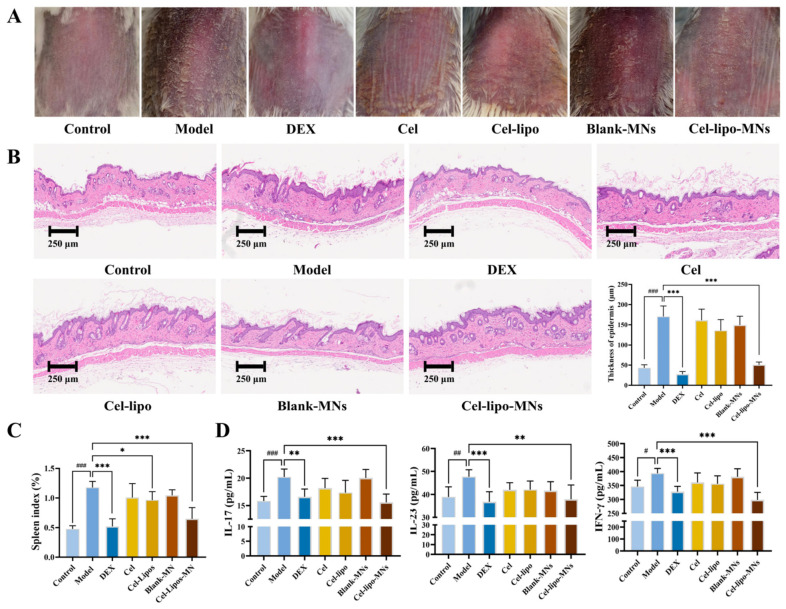
Evaluation of the therapeutic effects of Cel-lipo-MNs on psoriasis models. (**A**). Photographs of the dorsal skin of mice treated with different groups; (**B**). H&E staining and Epidermal thickness of mouse skin sections; (**C**). Spleen index of mice treated with different groups; (**D**). Expression levels of IL-17, IL-23 and IFN-γ in the serum of psoriasis mice from different formulation groups. Data are expressed as mean ± SD (*n* = 6). * or # *p* < 0.05, ** or ## *p* < 0.01, and *** or ### *p* < 0.001. “#” stands for drug group vs. Control group, “*” stands for drug groups vs. Model group.

To further investigate the effects of drug intervention on the histopathological changes of psoriasis-like skin lesions, H&E staining was performed on the lesional dorsal skin tissues from each group of mice (Figure 4B). The skin of the control group showed an intact structure without obvious pathological abnormalities. In contrast, the model group exhibited typical histopathological changes, including marked hyperkeratosis and parakeratosis in the stratum corneum, thickening of the spinous layer accompanied by elongation of the rete ridges, as well as dilated and congested capillary loops in the dermal papillae [49]. Compared with the model group, the Cel-lipo-MNs group markedly ameliorated keratinization abnormalities and overall histopathological damage (*p* < 0.001), with a therapeutic effect obviously superior to that of the Cel and Cel-lipo groups. Epidermal thickening caused by abnormal keratinocyte proliferation is an important histopathological hallmark of psoriasis. Therefore, epidermal thickness in the H&E-stained sections was further quantified using ImageJ. The results showed that epidermal thickness was significantly increased in the model group compared with the control group. In contrast, both the DEX group and Cel-lipo-MNs group significantly reduced epidermal thickness relative to the model group (*p* < 0.001), whereas the Cel, Cel-lipo, and Blank-MNs groups only exhibited a downward trend without statistical significance. These results indicate that the Cel-lipo-loaded hydrogel microneedles can significantly inhibit psoriasis-like epidermal hyperplasia and improve skin histopathological structure.

In addition to local skin lesions, IMQ-induced psoriasis-like mouse models are often accompanied by systemic inflammatory responses and splenomegaly, resulting in an increased spleen index (spleen weight/body weight) [50]. As shown in Figure 4C, the spleen index of mice in the Model group was significantly elevated compared with that in the Control group, indicating that IMQ stimulation not only induced local psoriasis-like lesions, but also triggered a pronounced systemic immune-inflammatory response. Compared with the Model group, both the DEX group and the Cel-lipo-MNs group significantly reduced the spleen index (*p* < 0.001), suggesting that both treatments effectively alleviated the systemic inflammatory response associated with the model. In addition, the Cel-lipo group also significantly reduced the spleen index (*p* < 0.05), implying that liposomal encapsulation may enhance the anti-inflammatory and immunomodulatory effects of Cel by promoting its skin penetration and local delivery. In contrast, although the Cel group showed a downward trend, no statistically significant difference was observed. Overall, celastrol-loaded liposomal microneedles not only improved local psoriasis-like skin lesions, but also alleviated the systemic immune-inflammatory state of model mice to a certain extent, demonstrating favorable comprehensive therapeutic potential.

IL-23 is mainly secreted by dendritic cells and other immune cells, and promotes the expansion and stabilization of pathogenic Th17 cells, thereby sustaining chronic inflammatory responses. As a key effector cytokine, IL-17 can directly act on keratinocytes, inducing abnormal proliferation, differentiation imbalance, and the release of inflammatory mediators, which in turn drive typical pathological changes such as erythema, scaling, and epidermal thickening. Meanwhile, IFN-γ, as an important representative cytokine of Th1-type inflammation, can further enhance antigen presentation, inflammatory cell activation, and amplification of local immune responses, reflecting a broader disturbance of the immune network in psoriasis. Serum biochemical analysis (Figure 4D) showed that, compared with the Control group, the levels of IL-17, IL-23, and IFN-γ in the Model group were significantly increased (*p* < 0.001, *p* < 0.01, and *p* < 0.05, respectively), indicating that IMQ successfully induced a typical psoriasis-like immune-inflammatory response. Compared with the Model group, Cel-lipo-MNs significantly reduced the levels of these inflammatory cytokines (*p* < 0.001, *p* < 0.01, and *p* < 0.001, respectively), whereas the Cel, Cel-lipo, and Blank-MNs groups only showed downward trends without statistical significance. The superior therapeutic effect of Cel-lipo-MNs over free Cel and conventional liposomes may be attributed to microneedle-mediated trans-stratum corneum delivery, as well as the improved solubility, stability, and local retention of Cel provided by the liposomal formulation.

Overall, Cel-lipo-MNs exhibited a pronounced therapeutic effect against IMQ-induced psoriasis-like skin lesions, effectively alleviating erythema, scaling, and skin thickening, improving epidermal hyperplasia, and significantly suppressing IL-23/IL-17 axis- and IFN-γ-related inflammatory responses. Its overall therapeutic efficacy was superior to that of the free drug, conventional liposomes, and blank microneedles, demonstrating promising potential for local anti-psoriatic application.

### 3.2. Formatting of Mathematical Components


(1) $$Hemolysis(\%)=\frac{OD_{sample}-OD_{negative}}{OD_{positive}-OD_{negative}}\times 100\%$$

## 4. Discussion

In the present study, a Cel-lipo-MNs system was successfully developed by integrating liposomal nanocarriers loaded Cel with a photocrosslinked PEGDA-AM hydrogel microneedle platform. The obtained Cel-lipo exhibited a relatively uniform nanoscale particle size (~150 nm), high encapsulation efficiency. Importantly, the low zeta potential observed in the optimized Cel-lipo, which may be attributed to the DSPE-PEG2000 used in our work is negatively charged and may partially neutralize the charge of the positively charged liposome components [51], thereby showing a reduced zeta potential. Following a one-month observation period, the Cel-lipo system demonstrated good long-term stability, thereby addressing, to some extent, concerns regarding stability arising from low zeta potential. Although the Cel-Lipo exhibited favorable characteristics in terms of size, PdI, and short-term storage stability, the independent effects of individual component ratios on these attributes were not systematically investigated. Future studies will employ a multi-parameter optimization approach, incorporating size, polydispersity, and stability as joint optimization endpoints, to further refine the liposome composition and enhance the robustness of the formulation.

Psoriasis is characterized by abnormal keratinocyte proliferation and excessive epidermal hyperplasia. Effective penetration of the stratum corneum is a prerequisite for transdermal drug delivery by microneedles in psoriasis [52]. Skin insertion studies further confirmed the successful penetration capability of Cel-lipo-MNs. Histological analysis revealed the formation of distinct microchannels extending into the epidermal regions after microneedle insertion. Notably, these penetration depths were highly relevant to the pathological distribution of psoriatic lesions, as inflammatory cell infiltration and keratinocyte hyperproliferation primarily occur within these skin layers. Moreover, the micropores induced by microneedle insertion gradually recovered within a short period, indicating that the skin disruption caused by Cel-lipo-MNs was transient and minimally invasive. These findings suggest that the developed microneedle system could effectively penetrate the stratum corneum barrier while maintaining favorable skin safety.

The therapeutic efficacy of Cel-lipo-MNs was systematically evaluated in an IMQ-induced psoriasis-like mouse model, which is one of the most widely used psoriasis-like models and can rapidly induce typical features resembling human psoriasis, including erythema, scaling, and skin thickening [53]. In the present study, IMQ-treated mice developed typical psoriasis-like symptoms, including erythema, scaling, epidermal thickening, and increased PASI-like scores, confirming successful model establishment. Compared with the model group, Cel-lipo-MNs markedly improved the psoriasis-like lesions, acanthosis, and abnormal epidermal hyperplasia. Interestingly, although Cel-lipo alone exhibited partial improvement in splenic inflammation, its therapeutic effect on local skin lesions and inflammatory cytokine regulation remained limited compared with Cel-lipo-MNs.

Moreover, the anti-psoriatic efficacy of Cel-lipo-MNs was further evidenced by the significant reduction in the serum levels of IL-17, IL-23, and IFN-γ. These cytokines represent pivotal nodes in the complex inflammatory network of psoriasis: IL-23, predominantly secreted by dendritic cells and other immune cells, drives the expansion and stabilization of pathogenic Th17 cells, which in turn produce IL-17—a key effector cytokine that directly acts on keratinocytes to induce abnormal proliferation, differentiation imbalance, and release of inflammatory mediators, thereby causing erythema, scaling, and epidermal thickening. IFN-γ, a representative Th1-type cytokine, further amplifies local immune responses by enhancing antigen presentation and inflammatory cell activation. The fact that Cel-lipo-MNs markedly suppressed all three cytokines, whereas free celastrol, conventional liposomes, and blank MNs only exhibited trends without statistical significance, underscores the superiority of the integrated microneedle–liposome system. These findings suggest that the therapeutic effects of Cel-lipo-MNs may involve suppression of the IL-23/IL-17 inflammatory axis [4] and modulation of broader immune-inflammatory networks. The treatment may inhibit RORγt expression and decreased γδT cell recruitment, the latter being a primary source of IL-17 in psoriatic inflammation [54].

The superior anti-psoriatic efficacy of Cel-lipo-MNs may be attributed to several factors. First, microneedle-assisted delivery bypasses the stratum corneum barrier and increases drug deposition within diseased skin tissue. Second, liposomal encapsulation improves the solubility and local retention of Cel. Third, the hydrogel matrix may provide sustained release behavior through swelling-mediated diffusion, thereby prolonging local drug exposure.

Although the present study demonstrated promising anti-psoriatic efficacy of Cel-lipo-MNs, several limitations still remain. First, the long-term biosafety and repeated-administration tolerance of the PEGDA-AM hydrogel microneedles require further investigation. Second, the precise molecular mechanisms underlying the immunomodulatory effects of Cel-lipo-MNs remain incompletely understood and warrant additional mechanistic studies. Third, the present work mainly focused on acute IMQ-induced psoriasis-like lesions, while chronic and recurrent psoriasis models may better reflect clinical disease progression. In addition, optimization of drug release kinetics, skin retention behavior, and large-scale manufacturing processes should be further explored to facilitate future translational applications.

## 5. Conclusions

In this study, celastrol (Cel) was used as the active drug to construct a photocrosslinked hydrogel microneedle delivery system loaded with celastrol-containing liposomes (Cel-lipo-MNs). First, Cel-lipo was prepared by the thin-film dispersion method and its formulation was optimized, yielding liposomes with a uniform particle size distribution and favorable encapsulation performance. Subsequently, Cel-lipo-MNs were fabricated using a multistep vacuum micromolding process combined with UV-induced photocrosslinking. The resulting microneedles showed intact morphology, uniform needle structure, and favorable mechanical properties as well as skin insertion capability. Pharmacodynamic studies demonstrated that, Cel-lipo-MNs significantly alleviated typical psoriatic manifestations, reduced PASI-like scores, and mitigated body weight loss in model mice. Histopathological observation and quantitative analysis further showed that Cel-lipo-MNs markedly attenuated abnormal epidermal thickening and improved psoriasis-like pathological damage. The reduced serum levels of IL-17, IL-23, and IFN-γ suggested that the anti-psoriatic effect of Cel-lipo-MNs may be associated with regulation of the IL-23/IL-17 inflammatory axis and related immune-inflammatory networks. Overall, this study provided good experimental support and a potential research strategy for the development of transdermal formulations for psoriasis treatment.

## Data Availability

Data will be made available on request.

## Abbreviations

The following abbreviations are used in this manuscript:

Cel	Celastrol
Cel-lipo	Celastrol-loaded liposomes
Cel-lipo-MNs	Photocrosslinked hydrogel microneedles loaded with Cel-lipo
DSPE-PEG2000	Distearoyl phosphatidylethanolamine-polyethylene glycol 2000
AM	Acrylamide
PEGDA	Polyethylene glycol diacrylate
I2959	2-hydroxy-4’-(2-hydroxyethoxy)-2-methylpropiophenone
FTIR	Fourier transform infrared
IMQ	Imiquimod
IL-17	Interleukin-17
IL-23	Interleukin-23
IFN-γ	Interferon-gamma
PDI	Polydispersity index

## Appendix A

### Optimization of the Cel-Lipo Formulation

One-factor experiments were conducted using the mass ratio of drug to total lipids (1:5, 1:8, and 1:10), the content of DSPE-PEG2000 (5%, 10%, and 15%), and ultrasonication time (5, 10, and 15 min) as independent variables. Based on the results (Figure A1), the final Cel-Lipo formulation was determined to have a drug-to-total lipid mass ratio of 1:8, a DSPE-PEG2000 content of 15%, and an ultrasonication time of 10 min.
